# Homœopathic Prescription

**Published:** 1890-05

**Authors:** 


HOMŒOPATHIC PRESCRIPTION. A conversation by M. A. A. Wolff, M. D., Gainesville, Texas.

This is a tract upon Homœopathy, originally published as an article contributed to *The People's Journal of Health.* It was so much liked that the author was induced to publish it as a tract. It is written in the manner of a dialogue between a doctor and his patient. Doctors who wish to hand it around among their patients can have it in quantity, with their professional card attached, for $4.50 per thousand, by applying to the printer, George T. Yates, Gainesville, Texas.

